# Metagenomic Analysis of Microbial Diversity in Landfill Lysimeter Soil of Ghazipur Landfill Site, New Delhi, India

**DOI:** 10.1128/genomeA.01104-17

**Published:** 2017-10-19

**Authors:** Juhi Gupta, Rashmi Rathour, Madan Kumar, Indu Shekhar Thakur

**Affiliations:** School of Environmental Sciences, Jawaharlal Nehru University, New Delhi, India

## Abstract

We report the soil microbial diversity and functional aspects related to degradation of recalcitrant compounds, determined using a metagenomic approach, in a landfill lysimeter prepared with soil from Ghazipur landfill site, New Delhi, India. Metagenomic analysis revealed the presence and functional diversity of complex microbial communities responsible for waste degradation.

## GENOME ANNOUNCEMENT

Soil provides an intricate and challenging environment for microbiologists, with a large percentage of uncharacterized microbes that can be elucidated only through a metagenomic approach (1, 2). A lysimeter is a simulation of existing landfill conditions for waste degradation and leachate treatment that helps to resolve the problems associated with unengineered waste dumping sites (3). The aim of this study was to investigate through a metagenomic approach the functional genetic diversity of the diverse microbial communities responsible for degradation in a landfill lysimeter. Samples were collected in August 2016 from a landfill site (28°37′25.11″N, 77°19′36.1″E) located at the Ghazipur area in New Delhi, India. A composite sample was prepared from sampling positions and transferred to the physical setup of a lysimeter to study the leachate characteristics in an open dump.

DNA was extracted from a landfill lysimeter soil sample using an Exgene soil DNA kit (GeneAll Biotechnology Co., Ltd.). Paired-end sequencing libraries (2 × 150 bp) were prepared using an Illumina TruSeq Nano DNA library pep kit followed by sequencing using the Illumina NextSeq 500 platform. The raw reads were processed through Trimmomatic v 0.35 (4) to obtain high-quality clean reads devoid of adapter sequences and low-quality reads. The high-quality reads were assembled through CLC Genomics Workbench. Prodigal 2.6.3 (5) was used with default parameters to predict genes from the assembled scaffolds. Kaiju (6) (a program for sensitive taxonomic classification of high-throughput metagenomic sequencing data) was used for taxonomical analysis of the predicted genes, and functional analysis of genes from the sample was carried out using COGNIZER (7) (a comprehensive standalone framework) enabled to simultaneously provide COG (8), KEGG (9), Pfam (10), GO (11), and SEED (12) subsystems annotation to individual sequences constituting metagenomic data sets.

A mean library fragment size of 490 bp and ∼3 Gb high-quality data were obtained. A total of 10,254,512 high-quality reads were assembled into scaffolds. A scaffold of 406,004 bp was retrieved, with an *N*_50_ value of 454 bp. The software predicted a total of 258,260 genes with an average length of 337 bp. The predicted genes with <300 bp were excluded from taxonomical analysis and functional classification. Taxonomical classification was performed, and the results consisted of 65.80% bacteria, 2.93% *Archaea*, 0.92% eukaryotes, 0.13% viruses, and 30.23% unclassified organisms. The phylum *Proteobacteria*, with a percentage abundance of 28.24%, was found to be the most dominant phylum. The other major phyla and percentage abundances were *Actinobacteria* 9.71%, *Bacteroidetes* 4.66%, *Chloroflexi* 3.87%, *Firmicutes* 2.47%, *Gemmatimonadetes* 2.09%, *Planctomycetes* 1.61%, *Acidobacteria* 1.52%, *Ignavibacteriae* 1.51%, *Euryarchaeota* 1.31%, *Thaumarchaeota* 1.21%, *Cyanobacteria* 1.13%, “*Candidatus* Woesebacteria” 0.58%, *Deinococcus-Thermus* 0.52%, *Ascomycota* 0.35%, *Nitrospirae* 0.35%, *Verrucomicrobia* 0.34%, and *Balneolaeota* 0.30%. Other phyla were present in much lower percentages. At the genus level, the *Streptomyces* genus, with a percentage abundance of 1.85%, was found to be the most abundant. During the functional analysis of the metagenome, the leading pathway subclasses found included those involving metabolism of carbohydrate, amino acids, energy, nucleotides, and lipids, xenobiotic biodegradation.

The metagenomic analysis revealed the presence of diverse microbial communities and the functional analysis suggested the presence of various genes related to degradation of xenobiotic compounds under prevailing environmental conditions.

### Accession number(s).

The obtained nucleotide sequences were submitted to the NCBI Sequence Read Archive (SRA) under the accession number SRX2861368.
